# Chronic Obstructive Pulmonary Disease (COPD) as a disease of early aging: Evidence from the EpiChron Cohort

**DOI:** 10.1371/journal.pone.0193143

**Published:** 2018-02-22

**Authors:** Miguel J. Divo, Bartolome R. Celli, Beatriz Poblador-Plou, Amaia Calderón-Larrañaga, Juan Pablo de-Torres, Luis A. Gimeno-Feliu, Juan Bertó, Javier J. Zulueta, Ciro Casanova, Victor M. Pinto-Plata, Carlos Cabrera-Lopez, Francesca Polverino, Jonás Carmona Píréz, Alexandra Prados-Torres, Jose M. Marin, on behalf of the EpiChron—BODE Collaborative Group

**Affiliations:** 1 Pulmonary and Critical Care Division, Brigham and Women’s Hospital, Harvard Medical School, Boston, Massachusetts, United States of America; 2 EpiChron Research Group on Chronic Diseases, Instituto Investigación Sanitaria Aragón (IISAragón), REDISSEC (ISCIII), Hospital Universitario Miguel Servet University Hospital, Zaragoza, Spain; 3 Pulmonary Department, Clínica Universidad de Navarra, Pamplona, Spain; 4 Pulmonary Department, Hospital Universitario La Candelaria, Universidad de La Laguna, Carretera del Rosario, Santa Cruz de Tenerife, Spain; 5 Pulmonary and Critical Care Division, Baystate Medical Center, Springfield, Massachusetts, United States of America; 6 Pulmonary Department, Hospital Universitario de Gran Canaria Dr Negrin, Las Palmas de Gran Canarias, Spain; 7 Pulmonary Department, Hospital Universitario Miguel Servet, IISAragón & CIBERES, Zaragoza, Spain; National and Kapodistrian University of Athens, GREECE

## Abstract

**Background:**

Aging is an important risk factor for most chronic diseases. Patients with COPD develop more comorbidities than non-COPD subjects. We hypothesized that the development of comorbidities characteristically affecting the elderly occur at an earlier age in subjects with the diagnosis of COPD.

**Methods and findings:**

We included all subjects carrying the diagnosis of COPD (n = 27,617), and a similar number of age and sex matched individuals without the diagnosis, extracted from the 727,241 records of individuals 40 years and older included in the EpiChron Cohort (Aragon, Spain). We compared the cumulative number of comorbidities, their prevalence and the mortality risk between both groups. Using network analysis, we explored the connectivity between comorbidities and the most influential comorbidities in both groups. We divided the groups into 5 incremental age categories and compared their comorbidity networks. We then selected those comorbidities known to affect primarily the elderly and compared their prevalence across the 5 age groups. In addition, we replicated the analysis in the smokers’ subgroup to correct for the confounding effect of cigarette smoking. Subjects with COPD had more comorbidities and died at a younger age compared to controls. Comparison of both cohorts across 5 incremental age groups showed that the number of comorbidities, the prevalence of diseases characteristic of aging and network’s density for the COPD group aged 56–65 were similar to those of non-COPD 15 to 20 years older. The findings persisted after adjusting for smoking.

**Conclusion:**

Multimorbidity increases with age but in patients carrying the diagnosis of COPD, these comorbidities are seen at an earlier age.

## Introduction

An increase in life expectancy has led to the aging of populations worldwide[1]. Aging is an important known risk factors for most chronic diseases, however there is variability in the burden of chronic diseases affecting the old[2,3]. Understanding this variability is key to differentiating pathologic from successful or normal aging[4]. The co-occurrence of chronic diseases is not a phenomenon of simple chance[5–7], but rather the expression of *complex* biological interactions between a susceptible individual and the cumulative effect of environmental exposures, differentially affecting body systems[8].

Chronic Obstructive Pulmonary Disease (COPD) is a representative model of a disease to help discern pathologic from normal aging[9]: COPD is a disease which affects millions of people[10], is the third leading cause of death worldwide [11], has a natural history that is relatively well understood[12] and frequently occurs with comorbidities[13,14]. Moreover, in a big data longitudinal study of the entire Danish population (6.2 million subjects), COPD was identified as a disease central to the progression of other chronic diseases[15].

COPD is thought to occur due to the slow, progressive and cumulative inflammatory response to inhaled particles, primarily from cigarette smoke and biomass fuel pollution. Such low grade persistent inflammation is a phenomenon associated with aging (thus the term “inflammaging”[16]), leading to the suggestion that COPD may actually represent a disease of accelerated aging[17,18].

Using network analysis, we tested the hypothesis that patients with a diagnosis of COPD would not only have an increased number of comorbidities characteristically seen in older individuals, but these would be detected at a younger age.

## Methods

### Study design and population

COPD cases and controls were selected from the EpiChron Cohort[19], which links, at the individual level, clinical and demographic information contained in the electronic medical records (EMR) of the 1.3 million inhabitants of the Spanish autonomous community of Aragon. The Spanish Health System is a single-payer, universal coverage, primary care based model, managed by the distinct autonomous communities[20]. The single, interconnected EMR system was implemented in the Aragon province in 2006 and since then each electronic medical record has accumulated past and new diagnoses generated from patient’s encounters including outpatient, hospital or emergency visit and from a proxy methods consisting of linking a specific diagnosis to every prescription and diagnostic. In this cross-sectional analysis, we included the anonymized records in the year 2011 of all individuals aged 40 years and older (n = 727,241), with a physician diagnosis of COPD and an equal number of age and gender matched group of subjects without the diagnosis. COPD cases were identified as those individuals carrying in their EMR any of the following International Classification of Diseases 9th Edition (ICD9) codes 490–492, 494 and 496, in at least two or more encounters[21,22]. We chose the year 2011 because it provides 5 years of cumulated experience since EMR implementation (2006) thus the records met the following standards: a percentage of uncoded episodes less than 20%, a percentage of notes (e.g., prescription, observation, etc.) listed in uncoded episodes less than 15%, a percentage of prescriptions linked to uncoded episodes less than 10%, and a percentage of patients with no diagnostic information less than 10%[23].

For each patient, demographics (age and gender), a list of chronic conditions accumulated over time and cumulatively recorded in the EMR were extracted using an algorithm validated in previous studies[23,24] and summarized in S1 Fig. In addition, we extracted admission(s) to acute care hospital for any reason in the prior year (2010) and mortality as of March 30th, 2014. We selected those diseases classified as chronic conditions based on the list proposed by Salisbury et al[25] totaling 119 diseases included in the final analyses (S1 Table).

### Statistical analysis

Data was summarized as mean and standard deviation (SD) or median and interquartile range (IQR) for continuous variables according to their distribution. Categorical or binomial variables are presented as proportions. We used two-sample t-tests, Wilcoxon-rank and Chi-square tests to compare groups, depending on the type of variable and their distribution. Comorbidities were tabulated as dichotomic variables representing the presence or absence of the disease. We compared the prevalence and the cumulative number of comorbidities per individual in the COPD and non-COPD cohorts. Multivariate analysis was used to evaluate the association between age and the cumulative number of comorbidities using COPD as the variable of interest. A logistic regression was used to determine the interaction between age, number of comorbidities and having the diagnosis of COPD is associated with death.

For hypothesis testing comparing both groups a p≤ 0.05 was deemed statistically significant whereas for Network construction where multiple correlation testing are performed a p≤ 0.01 was chosen as significant as published previously[5]. Finally, bootstrapping with 1000 replications was conducted to obtain the 95% confidence interval for the network’s statistics.

### Network construction and analysis

We constructed the network graphs applying the same methodology used in our previous work[5]and summarized in S2 Fig of the online supplement. For each network, we determined: 1. Network *density* or how many nodes and links hold the network together, 2. The median and interquartile range of *edges* for the nodes known as *degree*, 3. We plotted the *degree distribution* to determine if it follows a binomial or exponential distribution suggesting the network’s topology as *random or scale free*[26].4. Based on the *degree distribution*, we selected those nodes at the 75^th^ quartile representing the highly-connected nodes (*hubs*).

### Early aging

We divided both cohorts into 5 incremental age groups (between 40 and 55 years, 56 to 65, 66 to 75, 76 to 85 and above 85 years of age) and constructed their corresponding comorbidities networks to compare network’s density within and between age groups. Based on the criteria by Rowe and Kahn[27], we selected those diseases known to affect primarily the elderly and compared their prevalence at those age groups. The comorbidities included were cataracts, degenerative joint disease, diabetes mellitus, coronary artery disease, benign prostatic hypertrophy, osteoporosis, dementia, depression, hearing loss, skin and lung cancer, chronic renal failure and atherosclerosis. To discern if cigarette smoking alone and not COPD per se, has an independent effect on early aging[28], we conducted the same analysis by extracting the subgroup of confirmed smokers in the non-COPD population and compared the findings to a similar number of COPD patients matched for age and gender.

This study was approved by the Clinical Research Ethics Committee of Aragon (CEICA). All analyses were performed using JMP Pro ® software, version 12.0 (SAS Institute) and Gephi Graph software V-0·8·2 beta[29] to create and analyze the networks.

## Results

The age and gender distribution was similar in patients with the diagnosis of COPD and the controls as shown in Table 1.

**Table 1:** Subjects’ characteristics.

Demographics	COPD	Controls	p-value
Number	27,617	2,7617	
**Age brackets distribution (n, % of total for the group)**			
Age 40–55	3,267 (12%)	3,267 (12%)	
Age 56 to 65	5,169 (19%)	5,605 (20%)	
Age 66 to 75	7,768 (28%)	8,989 (33%)	
Age 76 to 85	8,764 (32%)	7,522 (27%)	
Age > 85	2,649 (10%)	2,234 (8%)	
**Gender (n, % of total for the group)**			
Male n (%)	19378 (70%)	19378 (70%)	
Female n (%)	8239 (30%)	8239 (30%)	
**Number of comorbidities per patient (Mean, SD, 95% CI)**			
Comorbidities (whole)	4.9 ± 3.4 (4.8–4.9)	3.1± 2.6 (3.1–3.2)	<0.001
Male	4.7 ± 3.3 (4.7–4.8)	3.0 ± 2.5 (2.9–3.0)	<0.001
Female	5.4 ± 3.6 (5.3–5.4)	3.5 ± 2.7 (3.5–3.6)	<0.001

### Comorbidity burden and outcomes

Excluding COPD in the count of co-existing diseases, patients with the diagnosis of COPD have an average of two or more comorbidities than the non-COPD patients and this difference is statistically significant (Table 1). In both groups, females had a higher number of comorbidities compared to males (p< 0.001).

**Fig 1:**
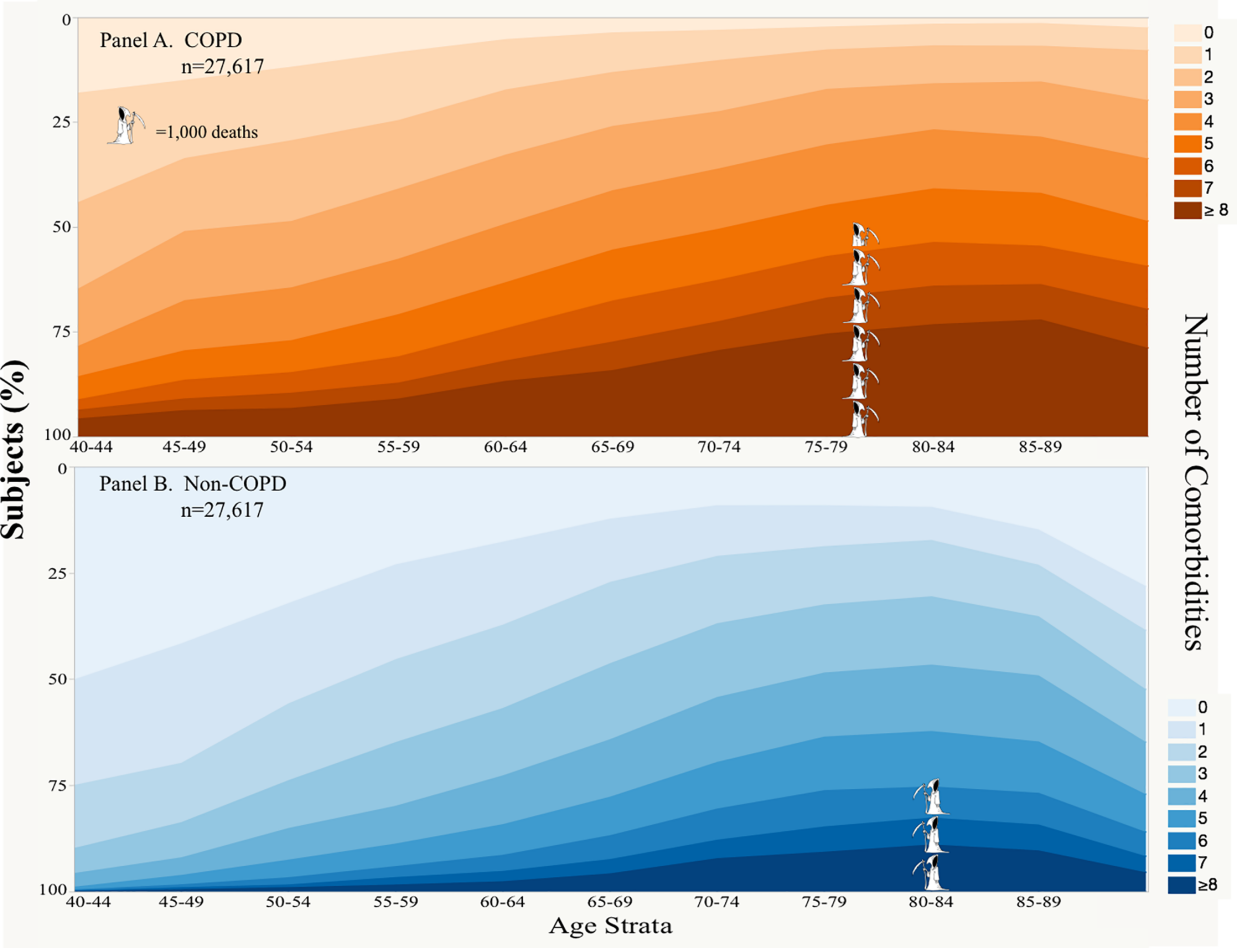
Number of comorbidities per individual by age brackets and total mortality. Each band represent the number of comorbidities/patient, and the width the proportion of individuals with that number of comorbidities at different age brackets (x-axis). Panel A represent those patients carrying the diagnosis of COPD and Panel B those without COPD. The stacked “Grim Reaper” represents the number of death at 3 years of follow-up (5,247 for COPD and 2,911 in the non-COPD) and their position is located at the mean age of death; one full “Grim Reaper” represents 1,000 deaths.

In both groups the proportion of patients with one or more comorbidity increased with age (Fig 1 and S3 Fig), however 50% of patients without the diagnosis of COPD at ages 40 to 44 years did not have any chronic disease while this occurred in only 18% of patients carrying the diagnosis of COPD (p<0.0001). Further, 55.9% of COPD diagnosed individuals aged between 40 and 44 years had two or more comorbidities. A similar proportion (54.7%) of non-COPD diagnosed individuals with two or more comorbidities occurred in the age group that was on average 15 years older (age 55 to 59) (Fig 1 Panels A and B, and S2 Table).

There were 5,247 deaths documented in the COPD cohort (19%) compared to 2,911 in the non-COPD (11%) group, and the difference is statistically significant (p< 0.001). In a logistic regression model age, number of comorbidities and carrying the diagnosis of COPD were significantly correlated with an increased risk for death (p< 0.001).

The odds ratio for 3-year mortality was 2.75 (95% CI 2.66–2.84) for every 10 years of age increase, 1.09 (95% CI 1.08–1.10) for every increment in the number of comorbidities, and 1.65 (95% CI 1.56–1.74) for carrying the diagnosis of COPD. There were 1,294 patients (4.6%) with the diagnosis of COPD admitted to a hospital during the year preceding study enrolment and 107 (0.3%) in the non-COPD cohort.

The prevalence of the 119 selected comorbidities ranged from 0.05 to 56%, and there was a significantly higher prevalence (p≤0.05) in ninety-nine of those comorbidities for patients carrying the diagnosis of COPD (S4 Fig).

### Chronic disease networks

The comorbidity networks are presented in S5 Fig Panel A for the COPD and Panel B for the non-COPD group. The non-COPD network is comprised of 118 nodes and 1685 links, whereas in the COPD group there are 119 nodes and 1917 links, resulting in a network with 232 more edges.

We plotted the node’s *degrees* (number of edges or links that a node possesses) distribution histograms (S6 Fig) and found a right-sided tailed distribution in both networks suggesting a scale-free architecture. The median number of links per node was 32 (IQR 16–46) for the COPD diagnosis group and 28 (IQR 13–38) for non-COPD. We identified those comorbidities that represent *hubs* by extracting those diseases with highest number of links, namely those in the upper quartile of the degree distribution histogram (S6 Fig). Thirty diseases (25% of all nodes) in each network met this definition. Out of those thirty diseases-hubs, twenty-four were similar in both groups. The six different diseases-hubs were: substance abuse, neuritis, cirrhosis, anxiety, aortic aneurism and obesity for the COPD network, and cerebrovascular disease, hyperlipidemia, dementia, eczema, cataracts and paralytic syndromes in the non-COPD network (S7 Fig and Table 2).

**Table 2:** Top connected diseases or hubs.

	**COPD**			**Non-COPD**		
**Rank**	Disease	Prevalence (%)	Degree (n)	Disease	Prevalence (%)	Degree (n)
1	Fe Def. Anemia	8	80	Arrhythmia	6.3	68
2	CVS NS	9.9	76	CVS NS	3.6	67
3	GERD	2.4	75	Fe Def. Anemia	3.9	67
4	Resp. Dis. NS	9.5	75	Resp. Dis. NS	1.2	66
5	Arrhythmia	14.4	68	HTN	46	64
6	HTN	55.6	65	CRF	0.8	62
7	Atherosclerosis	2.2	64	CHF	2.9	58
8	CHF	11.4	64	CAD	5.1	57
9	Depression	14.5	62	Diverticulosis	2	57
10	CRF	3.4	62	DM	16.8	56
11	Diverticulosis	4.2	61	DJD	19.7	55
12	CAD	9.7	57	Depression	9	55
13	Valvulopathy	4.9	56	Incontinence	5.2	54
14	Osteoporosis	10.5	55	Dyslipidemia	32.3	53
15	Hemat. Dis. NS	3.9	55	CVA	6.6	53
16	BPH	22.1	55	Atherosclerosis	0.4	52
17	DM	22.1	54	Hypothyroidism	5.5	52
18	Incontinence	9.1	54	GERD	0.5	52
19	Skin Ulcer	3.9	53	Hemat. Dis. NS	1.8	52
20	DJD	24.1	53	BPH	16.2	51
21	Neuritis	3.2	52	Varicose veins	11.1	49
22	Varicose veins	15.1	51	Neuro. Dis. NS	3.5	49
23	Endo. NS	3.2	51	Endo. NS	1.5	47
24	Cirrhosis	1.9	51	Valvulopathy	1.9	46
25	Neuro. Dis. NS	5.5	51	Dementia	3.4	46
26	Substance abuse	4.5	49	Osteoporosis	7.1	45
27	Hypothyroidism	7.9	48	Skin Ulcer	2	44
28	AA	0.7	46	Eczema	7.9	43
29	Obesity	15.9	46	Cataract	11.5	43
30	Anxiety	3.6	46	Paralytic synd.	0.3	42

Twenty-four disease representing network’s hubs are similar in both cohorts, however the ranking and prevalence differ in both groups. The colored cells represent hubs that differ between the two cohorts. Abbreviations: AA: Aortic Aneurism; BPH: Benign Prostatic Hypertrophy; CAD: Coronary Artery Disease; CHF: Congestive Heart Failure; CRF: Chronic renal failure; CVA: Cerebrovascular Accident; CVS NS: Other Cerebro Vascular Syndrome; DJD: Degenerative joint disease; DM: Diabetes Mellitus; Endo. NS: Other endocrinopathy; Fe Def. Anem.: Iron Deficiency Anemia; GERD: Gastro-Esophageal Reflux Disorder; Hemat. Dis. NS: Other Hematology Disorder; HTN: Hypertension; Neuro. Dis. NS: Neurologic Disorder, other; Resp. Dis. NS: Respiratory disorders, other;

### Accelerated aging

**Fig 2:**
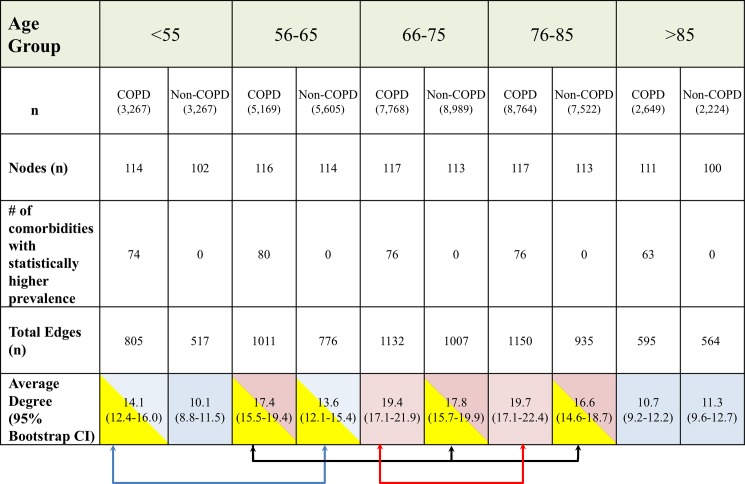
Comparison of network’s characteristics by 5-year age groups in both COPD and non-COPD groups. We categorized both groups into five age groups and built their respective networks to compare the network’s density: total number of nodes (3^rd^ row), total number of connections (4^th^ row), and average degree per node. Between groups comparisons were calculated for the average degree (last row) and those without statistical difference have similar color and relate to brackets.

Both cohorts were divided into five age groups as less than 55 years, 56 to 65 years, 66 to 75 years 76 to 85 and more than 85 years old. We constructed the comorbidities networks for each subgroup and compared each network’s density (number of nodes, number of edges and average degree) and diseases prevalence’s and summarized our findings in Fig 2. At every age category, the COPD Networks include more nodes, have a higher overall diseases prevalence and are much denser than the non-COPD, except for those older than 85 years old. When comparing networks density between age categories using the ANOVA test, there was a statistical difference between groups except for those shown in Fig 2 in which cells shaded with similar color and connected by brackets. This suggest that the distribution of network’s degree in the non-COPD aged 66 to 75 and 76 to 85 resemble that of the COPD at age 56 to 65.

### Comorbidities of aging

Table 3 shows that except for few instances, the prevalence of diseases related to aging are significantly higher in patients carrying the diagnosis of COPD within same age groups.

**Table 3:** Prevalence comparison of those diseases mostly occurring in elders.

		≤55 years	56–65 years	66–75 years	76–85 years	>85 years
**Degenerative Joint Disease**	Non-COPD	4.81%	13.38%	22.02%	27.05%	23.41%
	COPD	8.69%	18.48%	25.24%	30.53%	28.95%
	*Fisher’s exact test*	**< .0001**	**< .0001**	**< .0001**	**< .0001**	**< .0001**
**Atherosclerosis**	Non-COPD	0.00%	0.23%	0.33%	0.73%	1.12%
	COPD	0.43%	1.22%	2.09%	3.05%	3.55%
	*Fisher’s exact test*	**0.0001**	**< .0001**	**< .0001**	**< .0001**	**< .0001**
**Hearing Loss**	Non-COPD	2.82%	5.42%	7.03%	8.26%	7.34%
	COPD	4.81%	7.54%	8.55%	10.98%	11.55%
	*Fisher’s exact test*	**< .0001**	**< .0001**	**0.0003**	**< .0001**	**< .0001**
**Osteoporosis**	Non-COPD	1.93%	5.85%	7.87%	9.45%	6.58%
	COPD	3.95%	9.67%	11.10%	12.44%	12.31%
	*Fisher’s exact test*	**< .0001**	**< .0001**	**< .0001**	**< .0001**	**< .0001**
**Cataract**	Non-COPD	0.70%	3.76%	11.49%	20.58%	16.83%
	COPD	1.93%	6.73%	16.90%	25.22%	21.22%
	*Fisher’s exact test*	**< .0001**	**< .0001**	**< .0001**	**< .0001**	**0.0001**
**Benign Prostatic Hypertrophy**	Non-COPD	0.61%	6.05%	13.84%	16.46%	13.47%
	COPD	1.29%	8.03%	17.83%	22.39%	19.18%
	*Fisher’s exact test*	**0.0069**	**< .0001**	**< .0001**	**< .0001**	**< .0001**
**Skin CA**	Non-COPD	0.34%	0.93%	1.45%	2.52%	3.36%
	COPD	0.43%	1.08%	1.96%	3.19%	4.15%
	*Fisher’s exact test*	0.6895	0.4396	**0.0113**	**0.0111**	0.1534
**Lung CA**	Non-COPD	0.03%	0.27%	0.30%	0.27%	0.09%
	COPD	1.19%	2.09%	2.27%	1.32%	0.45%
	*Fisher’s exact test*	**< .0001**	**< .0001**	**< .0001**	**< .0001**	**0.0279**
**Dementia**	Non-COPD	0.18%	0.25%	1.78%	6.60%	11.32%
	COPD	0.43%	0.81%	2.95%	8.28%	14.76%
	*Fisher’s exact test*	0.1148	**< .0001**	**< .0001**	**< .0001**	**0.0004**
**Depression**	Non-COPD	7.35%	8.05%	9.04%	10.08%	10.03%
	COPD	16.19%	14.95%	12.40%	14.98%	15.89%
	*Fisher’s exact test*	**< .0001**	**< .0001**	**< .0001**	**< .0001**	**< .0001**
**Chronic Renal Failure**	Non-COPD	0.06%	0.20%	0.71%	1.25%	1.92%
	COPD	0.52%	1.06%	2.33%	5.33%	7.97%
	*Fisher’s exact test*	**0.0007**	**< .0001**	**< .0001**	**< .0001**	**< .0001**
**Coronary Artery Disease**	Non-COPD	0.80%	3.41%	5.51%	7.20%	6.76%
	COPD	3.46%	6.81%	10.16%	11.95%	13.82%
	*Fisher’s exact test*	**< .0001**	**< .0001**	**< .0001**	**< .0001**	**< .0001**

Comparison were made within the same age categories.

**Fig 3:**
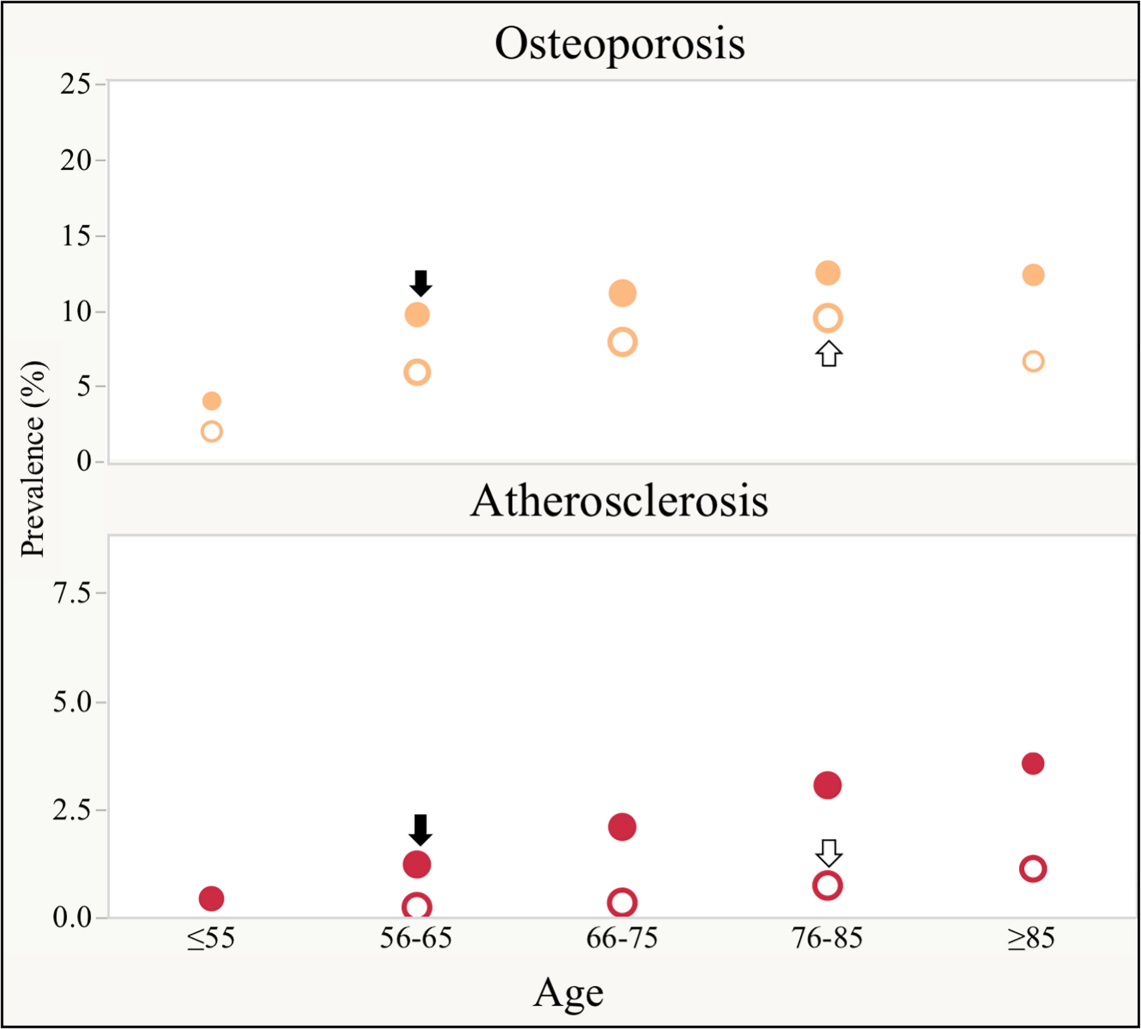
Prevalence of co-morbidities commonly seen in the elderly between COPD and non-COPD patients at five different age categories. Solid dots ● represent COPD patients and hollow dots ○ represent non-COPD patients. The size of the dots is proportional to the number of links (degrees) in their respective networks. Note, the prevalence (vertical axis) is higher in the COPD group and reach those values seen in non-COPD at an earlier age (horizontal axis). Comorbidities where the prevalence in COPD is similar to controls 10–20 years earlier are highlighted with a solid arrow ↓ and hollow arrow ⇓ in non-COPD. We show in this Fig 2 representative examples of comorbidities of the elder (osteoporosis and atherosclerosis). For the complete list of comorbidities seen in the elders please refer to S9 Fig.

Fig 3 on the other hand shows that similar prevalence can be observed in COPD patients aged between 56 and 65 years and those aged 76 to 85 years old in the non-COPD group (marked with arrows).

### Smokers subgroup analysis

There were 1,914 confirmed smokers in the non-COPD group and they were matched by age and gender with a similar number of randomly selected COPD patients. Their clinical characteristics are shown in S3 Table. In this subgroup analysis, COPD subjects had a higher number of comorbidities 4.5 ± 3.3 vs 3.3± 2.7 (p<0.001), accumulated the same number of comorbidities 15 years earlier than non-COPD smokers S8 Fig, had a higher absolute mortality (14% vs 7.4% p<0.001, S3 Table). As seen in the analysis of the entire cohort, the prevalence of age related comorbidities was higher and occurred at a younger age in COPD patients compared to *smokers* without COPD (S10 Fig).

## Discussion

There are three major findings in this study that compared age and sex matched subjects with and without the diagnosis of COPD extracted from single-payer, universal coverage population in Spain. First, the number and type of chronic conditions seen with aging, are registered at a younger age in subjects carrying the diagnosis of COPD. More notably, the density and comorbidity prevalence in the network of individuals carrying the diagnosis of COPD between the ages of 55 and 65 years resemble those of controls 10 to 20 years older. Second, the network analysis allowed us to identify thirty highly interconnected diseases or *hubs* in each group. Interestingly out of those thirty comorbidities, six were unique for each group. For the COPD cohort, these unique *hubs* represent mostly behavioral (substance abuse), lifestyle-metabolic (obesity) and psychiatric (anxiety) disorders which usually affect individuals at earlier stages of their life and perhaps are in part responsible for an early aging process (S7 Fig and Table 2). In contrast, for the non-COPD cohort the distinct *hubs* are diseases characteristically seen at later age (dementia, cerebrovascular accidents, cataracts, atherosclerosis). Third, in the subgroup analysis using non-COPD smokers, the same findings were observed, suggesting that pathologic aging associated with smoking is magnified in those who develop COPD. These three observations support the hypothesis that having the diagnosis of COPD is associated with early aging.

The process of *aging* is defined as the progressive decline in body function and homeostasis that occurs after the reproductive phase of life, leading to an increasing vulnerability to disability, multimorbidity and death[30,31]. Our study confirms the known increased vulnerability for comorbidities that comes with age, but emphasizes that carrying the diagnosis of COPD enhances this risk. Previous studies have suggested the presence of early aging in patients with COPD as manifested by the relationship between severity of airflow limitation and increased number and depth of facial wrinkles, stiffer peripheral arteries[32] and osteoporosis[33]. As shown in Fig 3 and S9 Fig, we expand on these observations by demonstrating that for diseases characteristically occurring in the elderly, the prevalence was higher in subjects with COPD compared with those without the disease. Importantly, a similar prevalence of those diseases was reached one or two decades earlier in patients with the diagnosis of COPD. The consequences were not menial since those patients carrying the diagnosis of COPD had more deaths and on average died at a younger age than patients without that diagnosis. Ito and Barnes proposed that the mechanisms leading to premature aging in COPD include cellular senescence, stem cell exhaustion, increased oxidative stress, alteration in the extracellular matrix, reduction in endogenous antiaging molecules and an alteration in protective pathways such as autophagy[17]. More recently, Rutten et al demonstrated that a panel of molecular markers of aging were distinctively altered in patients with COPD compared with non-obstructed current smokers and healthy control[34]. This suggests that patients with COPD have an enhanced pathobiological reaction to the exposure to cigarette smoke. Our findings, comparing the subgroup of smokers without COPD and those with COPD, provide evidence for this differential response to cigarette smoke and support the argument of differential susceptibility to accelerated aging among smokers. Using diseases networks, our results also suggest the possibility that organ systems, and not just the lungs, could be affected differentially by such aging related mechanisms and the phenotypic expression is the burden of comorbidities that co-occur beyond simple chance.

In addition, the network analysis allows us to better visualize the complex interactions between individual components and the identification of more influential nodes. This aggregate of multiple diseases and their statistical correlations provide the basis for the comorbidity network shown in Fig 3. It represents a complex system of diseases interacting to form an elaborate mesh of nodes and links, but this integrative methodology helps us discover two main emerging properties of the system hardly evident if we study individual or small groups of diseases separately. First, the comorbidity networks have features of scale-free networks, allowing us to identify *hubs* represented by those 25% of diseases holding more than 47% of all connections (S7 Fig and Table 2). Second, not surprisingly, 24 of the 30 diseases representing hubs were shared in both COPD and non-COPD networks. We identified, however, six other comorbidities unique to each group. Interestingly, for COPD, those unique *hubs* represent mostly behavioral (substance abuse), lifestyle (obesity) and psychiatric (anxiety) disorders which usually affect individuals at earlier stages of their life, and perhaps are in part responsible for the early aging process. As suggested in previous studies, those hubs could potentially be targeted for intervention as the basis of network perturbation experiments. This benefit of treatment cross-effect in multimorbidity is in dire need of testing[5,35–41].

The primary strength of this study is its large, real life, representative, primary care based population that enabled us to examine with precision the associations of a diverse and large group of chronic diseases affecting individuals at different age groups. However, as is true of population based studies, there are several limitations. First, the diagnosis of COPD could not be confirmed by spirometry leading to mis or underdiagnoses. To mitigate this limitation, we used the EpiChron database which is a clinical database rather than just an administrative one, we identified a large cohort of cases extracted from a broad primary and secondary interlinked practices, and used a validated algorithm to mine those cases and matched them. Mining electronic health records is becoming an increasingly relevant source of discovery[6,15,21,42] by providing large datasets, and in our study the unique opportunity to include 119 different chronic diseases. Arguably, a daunting and expensive task for a prospective study aimed to objectively confirm each of those conditions. Second, it is possible that the prevalence of current smokers was underestimated, as published prevalence estimate in Spain varies between 25–35% for adults 40 years and older[43], while in our sample smoking status was reported at a prevalence of 21% for COPD patients and 7% for non-COPD. To address this limitation, we conducted the same analysis on those confirmed smokers that had COPD and compared them with that of non-COPD subjects. The results were similar to those we observed in the whole cohort, indicating that the phenomenon of accelerated aging is more marked in susceptible smokers who develop COPD than in smokers in general. Third, we selected COPD as the index disease, nonetheless it is possible that our findings may not be exclusive of COPD, a point that will require future studies using other relevant chronic diseases. Finally, the study is cross-sectional by design limiting the possibility of exploring the evolution of disease networks over time. We included representative samples in all age categories, compared them with a control group and highlighted the within and the between age categories comparisons.

In conclusion, using comorbidities networks analysis, this study supports the concept that individuals carrying the diagnosis of COPD are more predisposed at an earlier age to diseases characteristically seen in the elderly. In addition, it highlights some influential comorbidities that could act as potential drivers of this process. Identifying these drivers should help clinicians screen for those diseases earlier in the clinical course of COPD patients. For the researchers, deciphering the pathobiological mechanisms underlying these interlinked diseases could result in interventions capable of preventing or reversing them.

## Supporting information

S1 Fig Chronic conditions extraction and classification methodology. a. ICPC: International Classification of Primary Care
b. ICD-9: International Statistical Classification of Diseases and Related Health Problems, version 9CG®: The Johns Hopkins Adjusted
c. Clinical Groups® System (The Johns Hopkins ACGH System (2008) Reference Manual Version 8.2)
(TIFF)

S2 Fig Network construction methodology and definitions. (TIFF)

S3 Fig Number of comorbidities per individual by age comparing COPD and Non-COPD regression. Regression plot of the relationship between the number of comorbidities per individual as a function of age comparing patients carrying the diagnosis of COPD and their matched controls. For patients carrying the diagnosis of COPD the regression equation is: Number of comorbidities = -0.17 + 0.07 x Age (years) and for non-COPD the equation is Number of comorbidities = -1.85 + 0·07 x Age (years).
(TIFF)

S4 Fig Comorbidity prevalence comparing COPD vs. non-COPD. Comorbidities with asterisk * denote a significantly higher prevalence (p< 0·05).
(PDF)

S5 Fig The COPD and non-COPD comorbidity networks. The size of the nodes is equivalent to their prevalence. The graphic layout is the result of a computational algorithm that considers the size of the nodes and the number, weight (Pearson correlation) and polarity (positive or negative correlation) of all links and places the most densely connected (hubs) and prevalent comorbidities in the center of the graph, while those comorbidities with less linkages are found in the periphery.
(TIFF)

S6 Fig Degree distribution histogram for both COPD and non-COPD networks. Degree is the number of edges or links that a node possesses (see example on the left panel). The histograms represent the distribution of the degrees for the 119 chronic diseases included in the networks. The upper panel belongs to the COPD network while the lower panel to the non-COPD network. The blue lines represent a density curve demonstrating a right-sided long tail and the red dotted line the 25^th^ and 75^th^ percentile distribution’s cutoffs. Highly connected diseases referred as “*hubs*” are in the upper quartiles.
(PDF)

S7 Fig Comorbidities sub-network representing the top connected diseases or hubs. The graph represents the subnetworks extracted from Fig 2 that include only those nodes (diseases) that represent network’s hubs. Nodes colored in red represents those hubs that are unique for each group (panel A for COPD and panel B for non-COPD). The size for the hubs are proportional to their degree (number of connections).
Abbreviations: AA: Aortic Aneurism, BPH: Benign Prostatic Hypertrophy, CAD: Coronary Artery Disease, CHF: Congestive Heart Failure, CRF: Chronic renal failure, CVA: Cerebrovascular Accident, CVS NS: Other Cerebro-Vascular Syndrome, DJD: Degenerative joint disease, DM: Diabetes Mellitus, Endo. NS: Other endocrinopathy, Fe Def. Anem.: Iron Deficiency Anemia, GERD: Gastro-Esophageal Reflux Disorder, Hemat. Dis. NS: Other Hematology Disorder, HTN: Hypertension, Neuro. Dis. NS: Neurologic Disorder non-specified, Resp. Dis. NS: Respiratory disorders non-specified.
(TIFF)

S8 Fig Number of comorbidities per individual by age comparing COPD and non-COPD regression with confirmed smoking status. (TIFF)

S9 Fig Prevalence of co-morbidities commonly seen in the elderly between COPD and non-COPD patients at five different age categories. Solid dots ● represent COPD patients and hollow dots ○ represent non-COPD patients. The size of the dots is proportional to the number of links (degrees) in their respective networks. Note, the prevalence (vertical axis) is higher in the COPD group and reach those values seen in non-COPD at an earlier age (horizontal axis). Comorbidities where the prevalence in COPD is similar to controls 10–20 years earlier are highlighted with a solid arrow ↓ and hollow arrow ⇓ in non-COPD. We show as representative example for this figure osteoporosis and atherosclerosis.
(TIFF)

S10 Fig Prevalence of co-morbidities commonly seen in the elderly between COPD and non-COPD *smokers* at five different age categories. (TIFF)

S1 Table List of chronic conditions included in the analysis. (DOCX)

S2 Table Number and percentage of individuals in each age categories distributed to their comorbidities load (0 to ≥8 comorbidities). Highlighted in green is the number and percentage of individuals aged 40 to 44 years with no comorbidities. For the non-COPD individuals 50% does not have any comorbidity, while those with the diagnosis of COPD is only 18%. In the red boxes, we are highlighting that more than 50% (55.9%) of COPD diagnosed individuals aged 40–45 have two or more comorbidities, while this occur for the non-COPD at ages 55 to 59. For all comparisons, the difference in proportions are statistically significant (p<0.0001).
(DOCX)

S3 Table Characteristics of the subgroup of confirmed smokers without COPD and their 1:1 random match. (DOCX)
