# Supplementary figures and images for: Chronic Obstructive Pulmonary Disease (COPD) as a disease of early aging: Evidence from the EpiChron Cohort
Source: PLoS One. 2018 Feb 22;13(2):e0193143. doi: 10.1371/journal.pone.0193143 (PMC5823454; doi:10.1371/journal.pone.0193143)

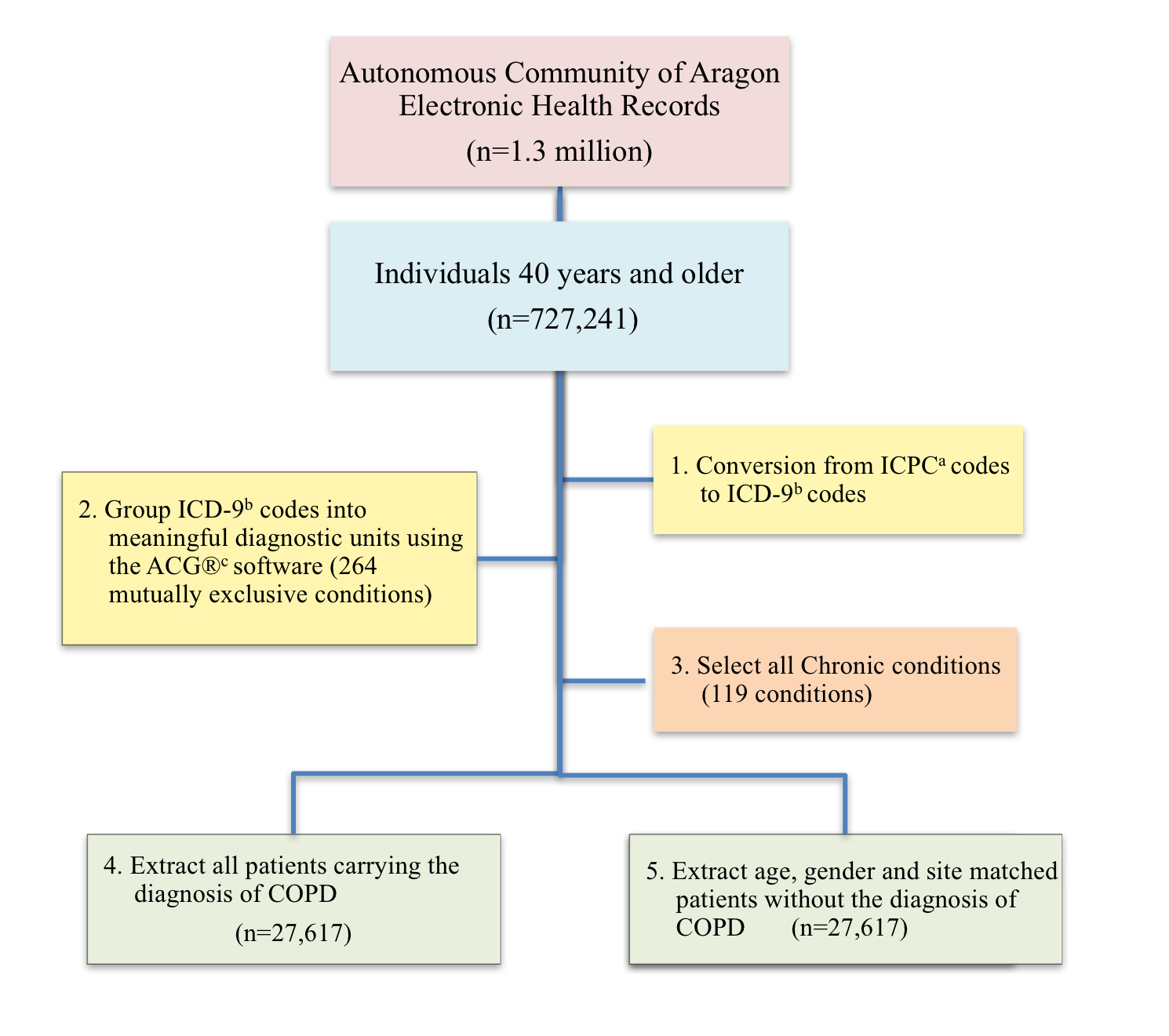

Supplement: S1 Fig — a. ICPC: International Classification of Primary Care b. ICD-9: International Statistical Classification of Diseases and Related Health Problems, version 9CG®: The Johns Hopkins Adjusted c. Clinical Groups® System (The Johns Hopkins ACGH System (2008) Reference Manual Version 8.2) (TIFF) [file pone.0193143.s001.tiff]

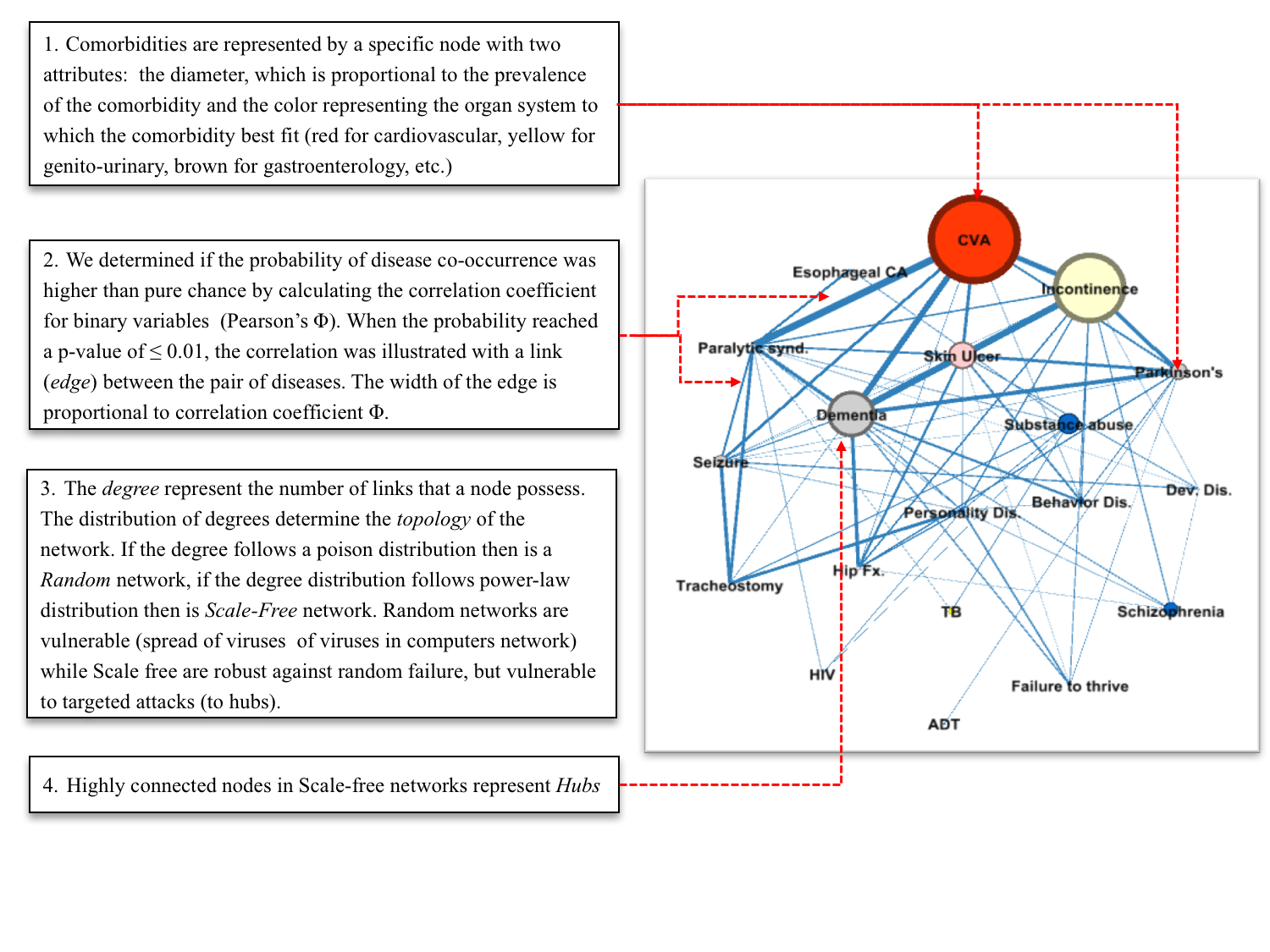

Supplement: S2 Fig — (TIFF) [file pone.0193143.s002.tiff]

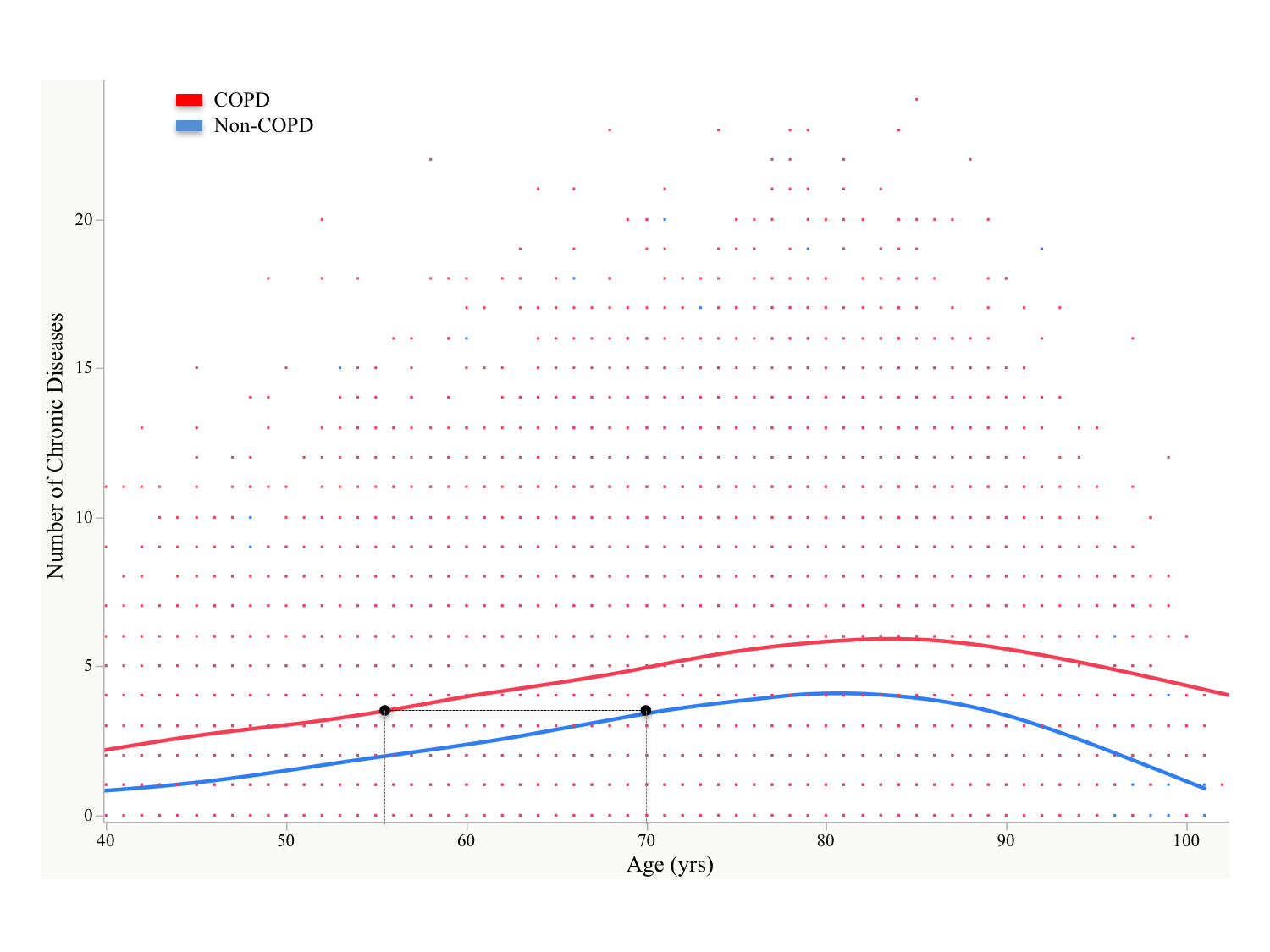

Supplement: S3 Fig — Regression plot of the relationship between the number of comorbidities per individual as a function of age comparing patients carrying the diagnosis of COPD and their matched controls. For patients carrying the diagnosis of COPD the regression equation is: Number of comorbidities = -0.17 + 0.07 x Age (years) and for non-COPD the equation is Number of comorbidities = -1.85 + 0·07 x Age (years). (TIFF) [file pone.0193143.s003.tiff]

Comorbidities prevalence comparison between COPD and Non-COPD patients

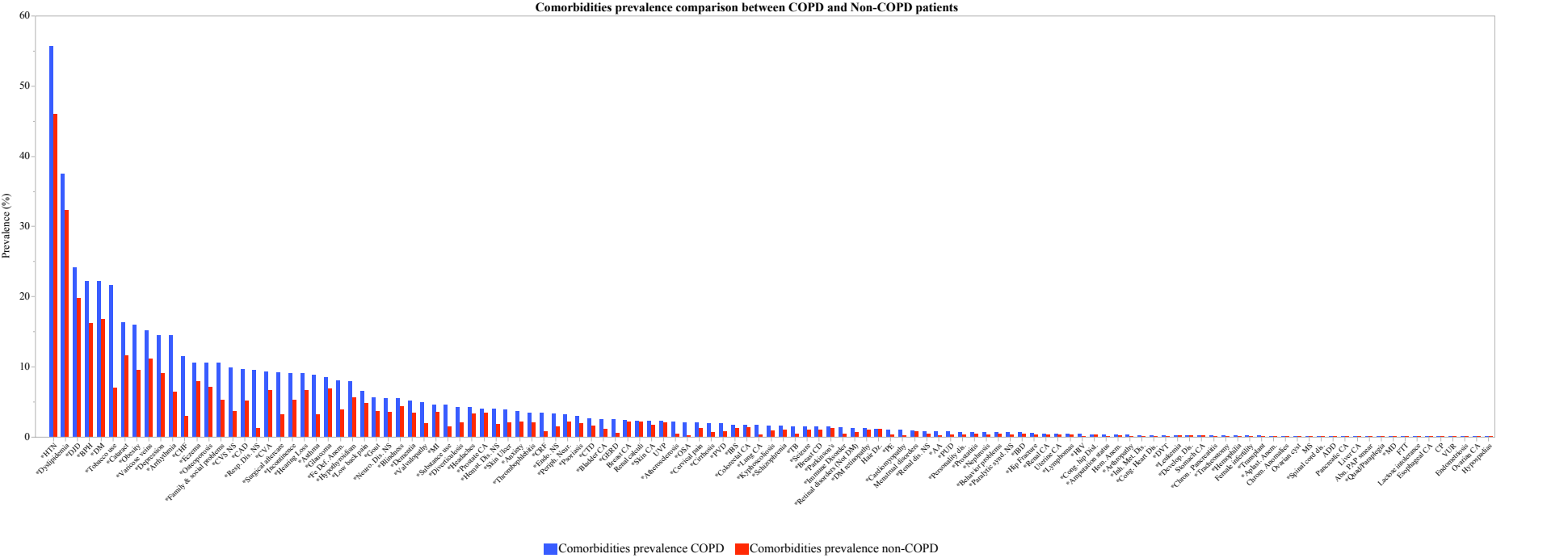

Supplement: S4 Fig — Comorbidities with asterisk * denote a significantly higher prevalence (p< 0·05). (PDF) [file pone.0193143.s004.pdf]

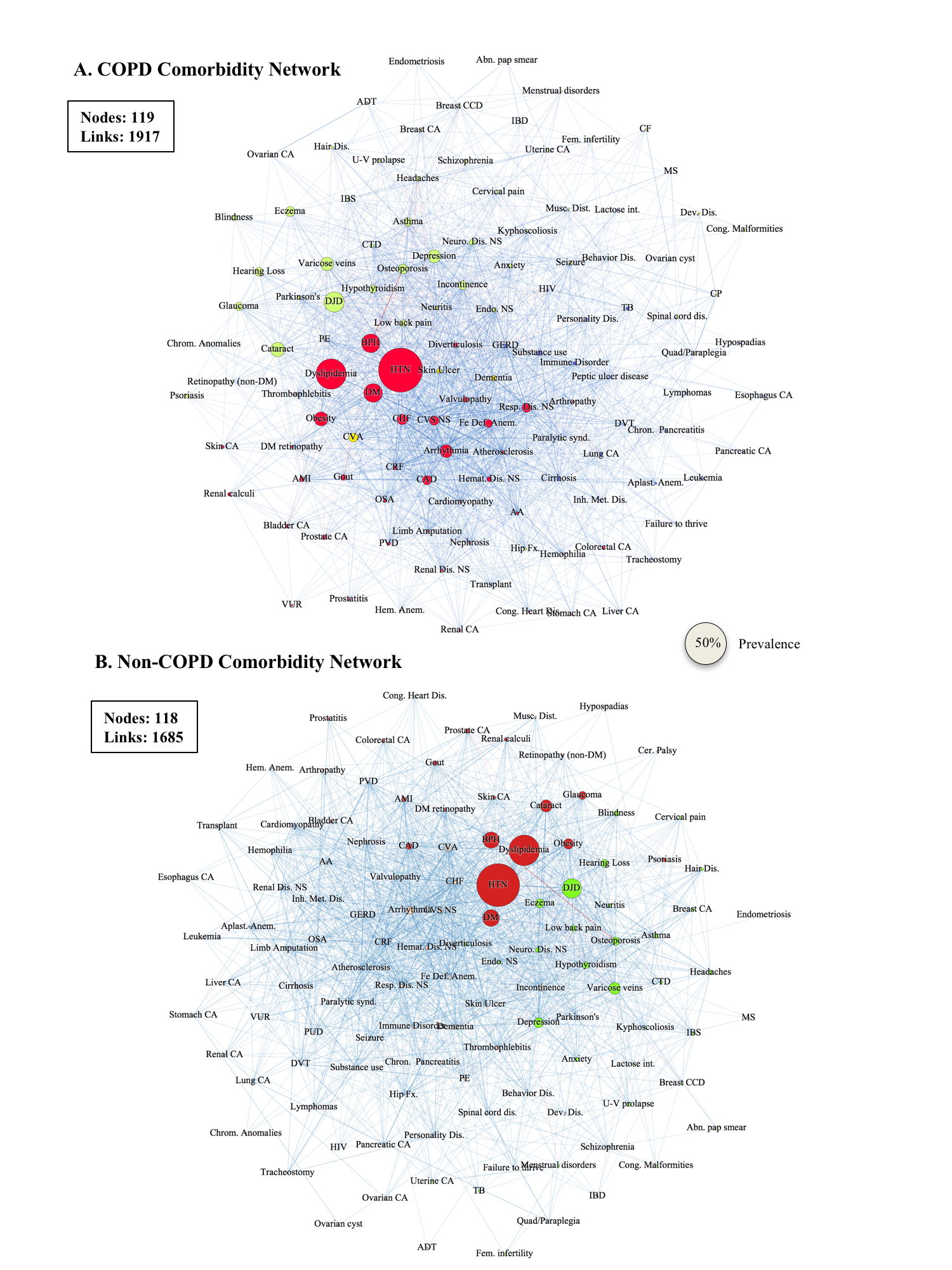

Supplement: S5 Fig — The size of the nodes is equivalent to their prevalence. The graphic layout is the result of a computational algorithm that considers the size of the nodes and the number, weight (Pearson correlation) and polarity (positive or negative correlation) of all links and places the most densely connected (hubs) and prevalent comorbidities in the center of the graph, while those comorbidities with less linkages are found in the periphery. (TIFF) [file pone.0193143.s005.tiff]

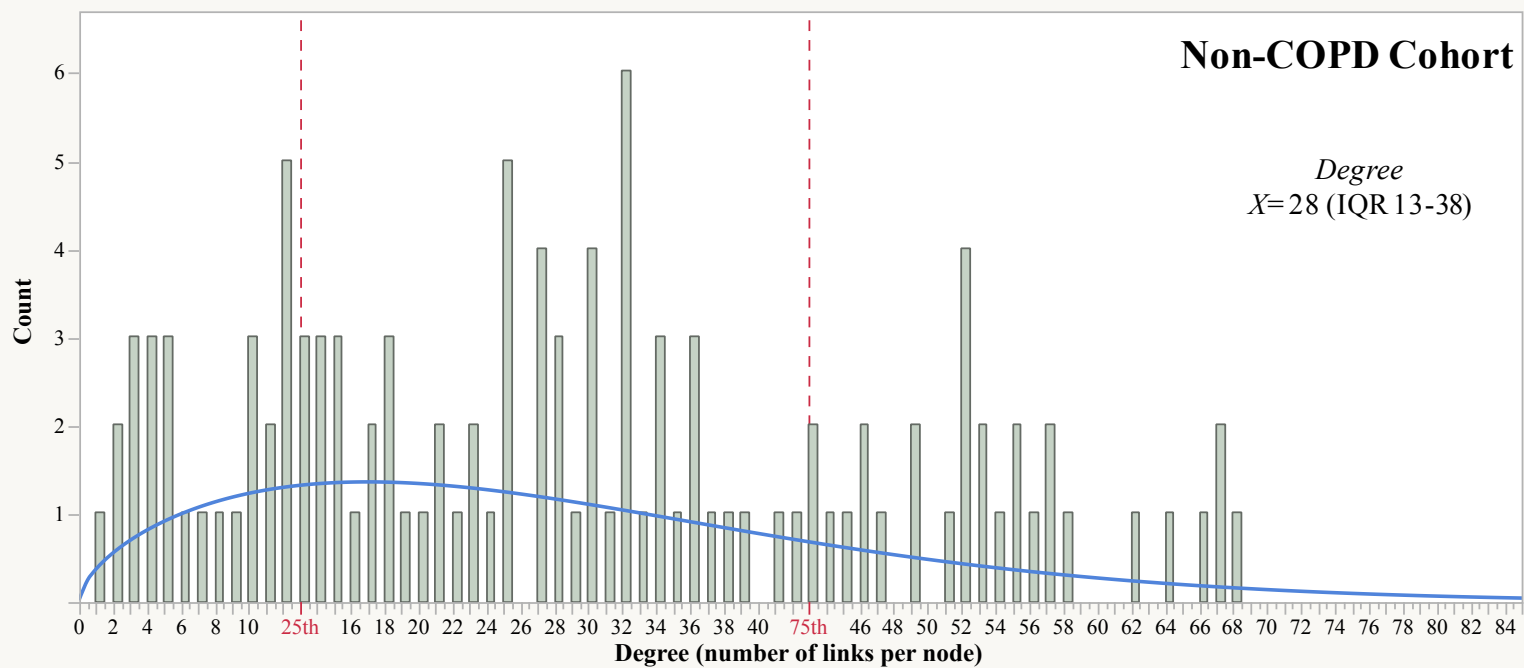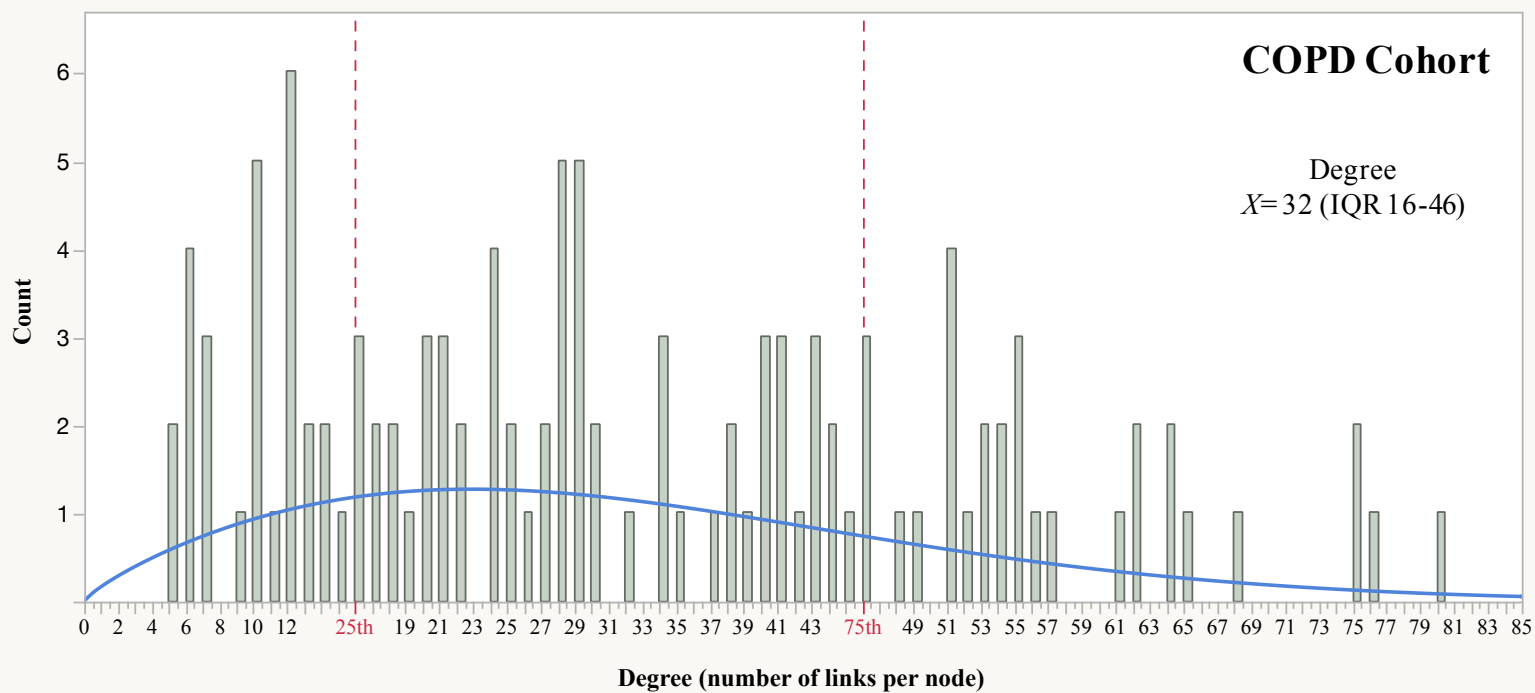

Supplement: S6 Fig — Degree is the number of edges or links that a node possesses (see example on the left panel). The histograms represent the distribution of the degrees for the 119 chronic diseases included in the networks. The upper panel belongs to the COPD network while the lower panel to the non-COPD network. The blue lines represent a density curve demonstrating a right-sided long tail and the red dotted line the 25th and 75th percentile distribution’s cutoffs. Highly connected diseases referred as “hubs” are in the upper quartiles. (PDF) [file pone.0193143.s006.pdf]

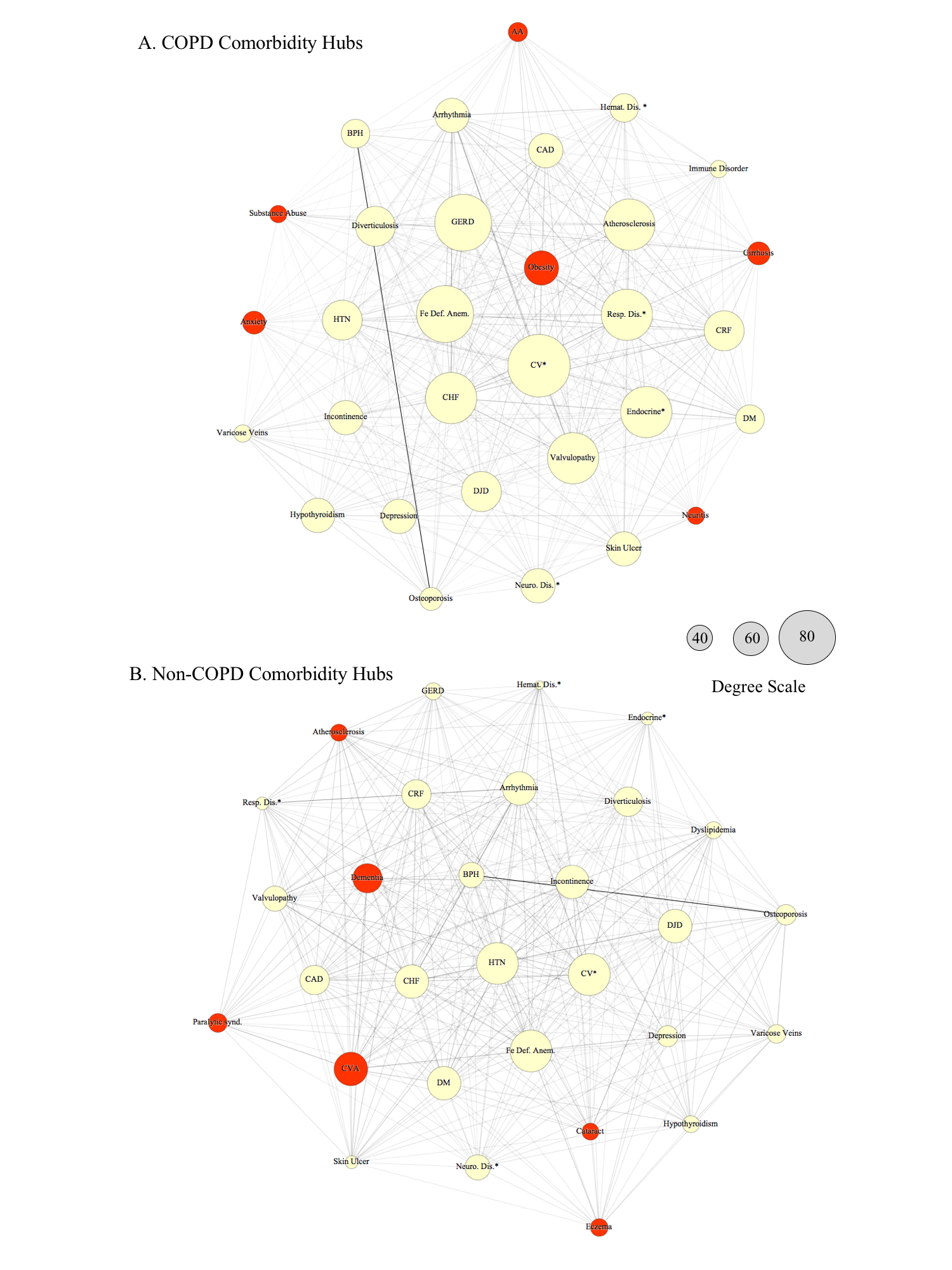

Supplement: S7 Fig — The graph represents the subnetworks extracted from Fig 2 that include only those nodes (diseases) that represent network’s hubs. Nodes colored in red represents those hubs that are unique for each group (panel A for COPD and panel B for non-COPD). The size for the hubs are proportional to their degree (number of connections). Abbreviations: AA: Aortic Aneurism, BPH: Benign Prostatic Hypertrophy, CAD: Coronary Artery Disease, CHF: Congestive Heart Failure, CRF: Chronic renal failure, CVA: Cerebrovascular Accident, CVS NS: Other Cerebro-Vascular Syndrome, DJD: Degenerative joint disease, DM: Diabetes Mellitus, Endo. NS: Other endocrinopathy, Fe Def. Anem.: Iron Deficiency Anemia, GERD: Gastro-Esophageal Reflux Disorder, Hemat. Dis. NS: Other Hematology Disorder, HTN: Hypertension, Neuro. Dis. NS: Neurologic Disorder non-specified, Resp. Dis. NS: Respiratory disorders non-specified. (TIFF) [file pone.0193143.s007.tiff]

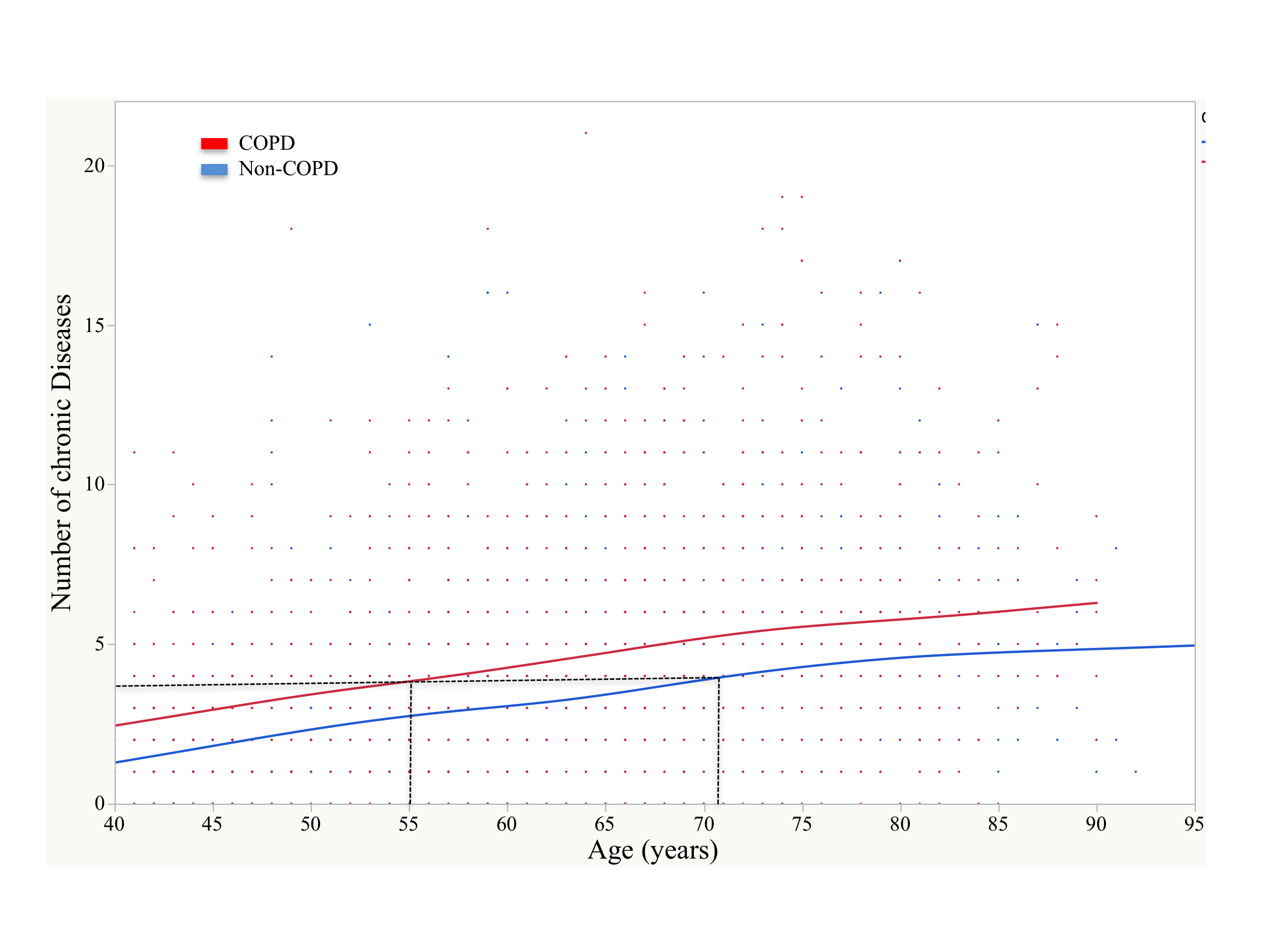

Supplement: S8 Fig — (TIFF) [file pone.0193143.s008.tiff]

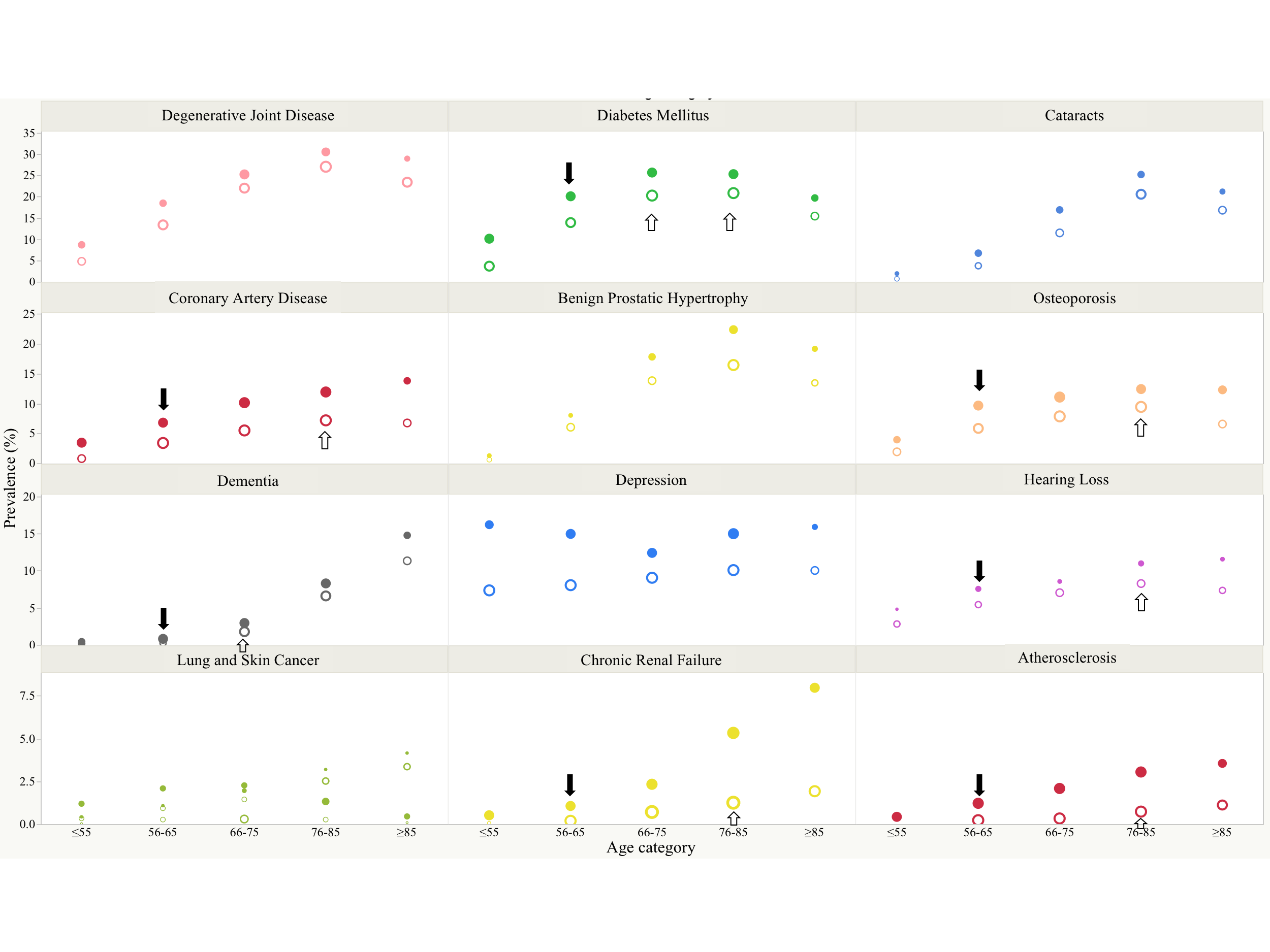

Supplement: S9 Fig — Solid dots ● represent COPD patients and hollow dots ○ represent non-COPD patients. The size of the dots is proportional to the number of links (degrees) in their respective networks. Note, the prevalence (vertical axis) is higher in the COPD group and reach those values seen in non-COPD at an earlier age (horizontal axis). Comorbidities where the prevalence in COPD is similar to controls 10–20 years earlier are highlighted with a solid arrow ↓ and hollow arrow ⇓ in non-COPD. We show as representative example for this figure osteoporosis and atherosclerosis. (TIFF) [file pone.0193143.s009.tiff]

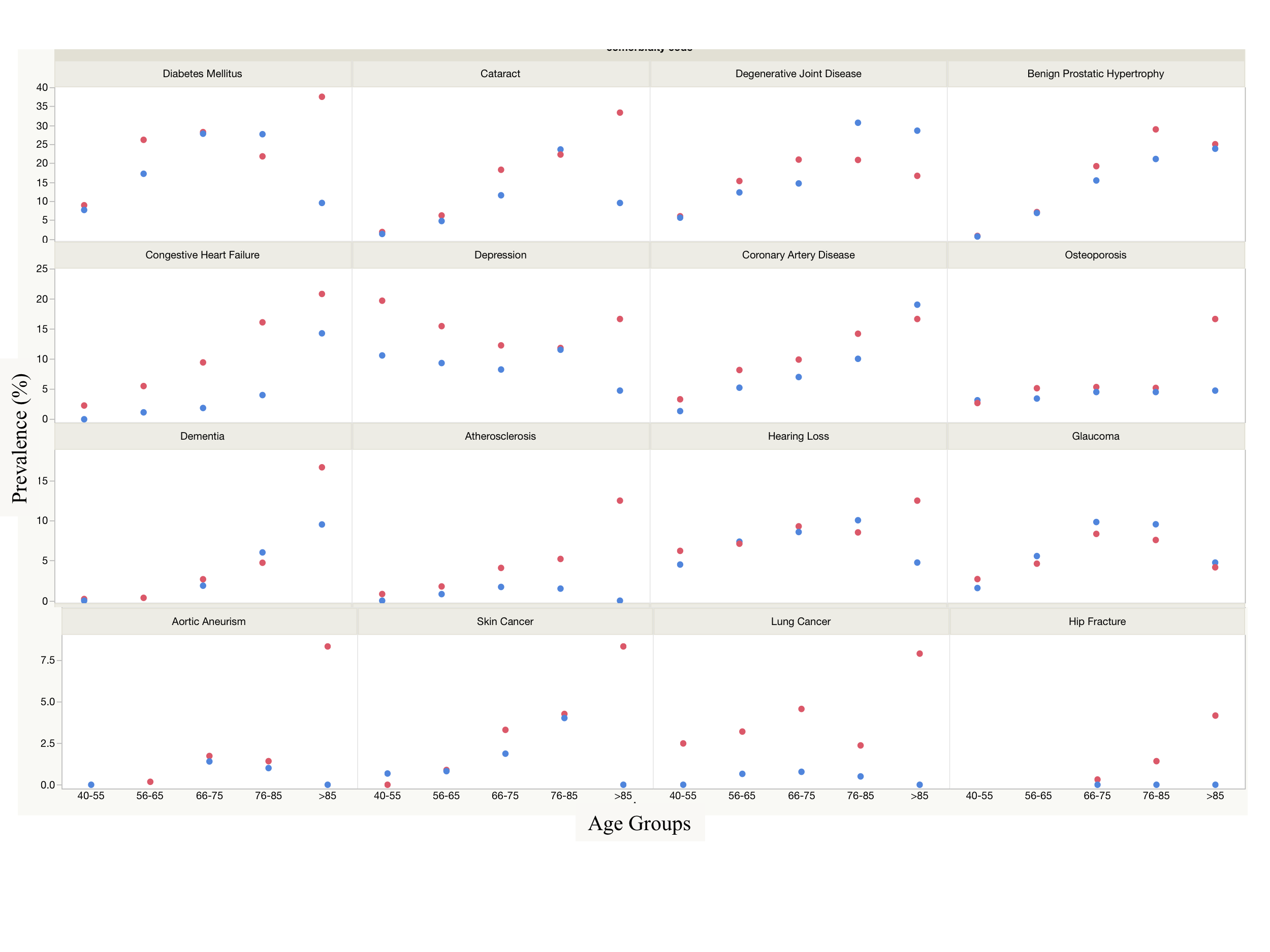

Supplement: S10 Fig — (TIFF) [file pone.0193143.s010.tiff]
