# Supplementary material for: Chronic Obstructive Pulmonary Disease (COPD) as a disease of early aging: Evidence from the EpiChron Cohort
Source: PLoS One. 2018 Feb 22;13(2):e0193143. doi: 10.1371/journal.pone.0193143 (PMC5823454; doi:10.1371/journal.pone.0193143)
Supplement: S1 Table — (DOCX) [file pone.0193143.s011.docx]

**S1 Table**

| **(EDC) Code** | **EDC Name** | **Node's Label** |
| --- | --- | --- |
| ADM02 | Surgical aftercare | Post. OP |
| ADM03 | Transplant status | Transplant |
| ALL06 | Disorders of the immune system | Immune Disorder |
| CAR03 | Ischemic heart disease (excluding acute myocardial infarction) | CAD |
| CAR04 | Congenital heart disease | Cong. Heart Dz. |
| CAR05 | Congestive heart failure | CHF |
| CAR06 | Cardiac valve disorders | Valvulopathy |
| CAR07 | Cardiomyopathy | Cardiomyopathy |
| CAR09 | Cardiac arrhythmia | Arrhythmia |
| CAR10 | Generalized atherosclerosis | Atherosclerosis |
| CAR11 | Disorders of lipid metabolism | Dyslipidemia |
| CAR12 | Acute myocardial infarction | AMI |
| CAR16 | Cardiovascular disorders, other | CVS NS |
| EAR08 | Deafness, hearing loss | Hearing Loss |
| END02 | Osteoporosis | Osteoporosis |
| END04 | Hypothyroidism | Hypothyroidism |
| END05 | Other endocrine disorders | Endo. NS |
| EYE02 | Blindness | Blindness |
| EYE03 | Retinal disorders | Retinal disorders (excluding diabetic retinopathy) |
| EYE06 | Cataract, aphakia | Cataract |
| EYE08 | Glaucoma | Glaucoma |
| EYE13 | Diabetic retinopathy | DM retinopathy |
| FRE03 | Endometriosis | Endometriosis |
| FRE12 | Utero-vaginal prolapse | Utero-vaginal prolapse |
| GAS02 | Inflammatory bowel disease | IBD |
| GAS05 | Chronic liver disease | Cirrhosis |
| GAS08 | Gastroesophageal reflux | GERD |
| GAS09 | Irritable bowel syndrome | IBS |
| GAS10 | Diverticular disease of colon | Diverticulosis |
| GAS12 | Chronic pancreatitis | Chron. Pancreatitis |
| GAS13 | Lactose intolerance | Lactose intolerance |
| GSU06 | Chronic cystic disease of the breast | Breast CCD |
| GSU08 | Varicose veins of lower extremities | Varicose veins |
| GSU11 | Peripheral vascular disease | PVD |
| GSU13 | Aortic aneurysm | AA |
| GTC01 | Chromosomal anomalies | Chrom. Anomalies |
| GTC02 | Inherited metabolic disorders | Inh. Met. Dis. |
| GUR01 | Vesicoureteral reflux | VUR |
| GUR03 | Hypospadias, other penile anomalies | Hypospadias |
| GUR04 | Prostatic hypertrophy | BPH |
| GUR09 | Renal calculi | Renal calculi |
| GUR10 | Prostatitis | Prostatitis |
| HEM01 | Hemolytic anemia | Hem. Anem. |
| HEM02 | Iron deficiency, other deficiency anemias | Fe Def. Anem. |
| HEM03 | Thrombophlebitis | Thrombophlebitis |
| HEM05 | Aplastic anemia | Aplast. Anem. |
| HEM07 | Hemophilia, coagulation disorder | Hemophilia |
| HEM08 | Hematologic disorders, other | Hemat. Dis. NS |
| INF01 | Tuberculosis infection | TB |
| INF04 | HIV, AIDS | HIV |
| MAL01 | Malignant neoplasms of the skin | Skin CA |
| MAL04 | Malignant neoplasms, breast | Breast CA |
| MAL05 | Malignant neoplasms, cervix, uterus | Uterine CA |
| MAL06 | Malignant neoplasms, ovary | Ovarian CA |
| MAL07 | Malignant neoplasms, esophagus | Esophagus CA |
| MAL08 | Malignant neoplasms, kidney | Renal CA |
| MAL09 | Malignant neoplasms, liver and biliary tract | Liver CA |
| MAL10 | Malignant neoplasms, lung | Lung CA |
| MAL11 | Malignant neoplasms, lymphomas | Lymphomas |
| MAL12 | Malignant neoplasms, colorectal | Colorectal CA |
| MAL13 | Malignant neoplasms, pancreas | Pancreatic CA |
| MAL14 | Malignant neoplasms, prostate | Prostate CA |
| MAL15 | Malignant neoplasms, stomach | Stomach CA |
| MAL16 | Acute leukemia | Leukemia |
| MAL18 | Malignant neoplasms, bladder | Bladder CA |
| MUS06 | Kyphoscoliosis | Kyphoscoliosis |
| MUS07 | Congenital hip dislocation | Cong. Hip Disloc. |
| MUS11 | Congenital anomalies of limbs, hands, and feet | Cong. Malformities |
| MUS13 | Cervical pain syndromes | Cervical pain |
| MUS14 | Low back pain | Low back pain |
| NUR03 | Peripheral neuropathy, neuritis | Neuritis |
| NUR05 | Cerebrovascular disease | CVA |
| NUR06 | Parkinson's disease | Parkinson's |
| NUR07 | Seizure disorder | Seizure |
| NUR08 | Multiple sclerosis | MS |
| NUR09 | Muscular dystrophy | MD |
| NUR11 | Dementia and delirium | Dementia |
| NUR12 | Quadriplegia and paraplegia | Quad/Paraplegia |
| NUR16 | Spinal cord injury/disorders | Spinal cord dis. |
| NUR17 | Paralytic syndromes, other | Paralytic synd. NS |
| NUR18 | Cerebral palsy | CP |
| NUR19 | Developmental disorder | Dev. Dis. |
| NUR21 | Neurologic disorders, other | Neuro. Dis. NS |
| NUT03 | Obesity | Obesity |
| PSY01 | Anxiety, neuroses | Anxiety |
| PSY02 | Substance use | Substance use |
| PSY04 | Behavior problems | Behavior problems |
| PSY05 | Attention deficit disorder | ADT |
| PSY07 | Schizophrenia and affective psychosis | Schizophrenia |
| PSY08 | Personality disorders | Personality dis. |
| PSY09 | Depression | Depression |
| REC01 | Cleft lip and palate | Cleft |
| REC03 | Chronic ulcer of the skin | Skin Ulcer |
| REN01 | Chronic renal failure | CRF |
| REN04 | Nephritis, nephrosis | Nephrosis |
| REN05 | Renal disorders, other | Renal dis. NS |
| RES03 | Cystic fibrosis | CF |
| RES04 | Emphysema, chronic bronchitis, COPD | COPD |
| RES06 | Sleep apnea | OSA |
| RES08 | Pulmonary embolism | PE |
| RES09 | Tracheostomy | Tracheostomy |
| RES11 | Respiratory disorders, other | Resp. Dis. NS |
| RHU01 | Autoimmune and connective tissue diseases | CTD |
| RHU02 | Gout | Gout |
| RHU03 | Arthropathy | Arthropathy |
| SKN02 | Dermatitis and eczema | Eczema |
| SKN12 | Psoriasis | Psoriasis |
| SKN13 | Disease of hair and hair follicles | Hair fol. Dz. |
