# Supplementary material for: Chronic Obstructive Pulmonary Disease (COPD) as a disease of early aging: Evidence from the EpiChron Cohort
Source: PLoS One. 2018 Feb 22;13(2):e0193143. doi: 10.1371/journal.pone.0193143 (PMC5823454; doi:10.1371/journal.pone.0193143)
Supplement: S2 Table — Highlighted in green is the number and percentage of individuals aged 40 to 44 years with no comorbidities. For the non-COPD individuals 50% does not have any comorbidity, while those with the diagnosis of COPD is only 18%. In the red boxes, we are highlighting that more than 50% (55.9%) of COPD diagnosed individuals aged 40–45 have two or more comorbidities, while this occur for the non-COPD at ages 55 to 59. For all comparisons, the difference in proportions are statistically significant (p<0.0001). (DOCX) [file pone.0193143.s012.docx]

| Age Category | 40-44 | | | | 45-49 | | | | 50-54 | | | | 55-59 | | | | 60-64 | | | | 65-69 | | | | 70-74 | | | | 75-79 | | | | 80-84 | | | | 85-89 | | | | >90 | | | |
| --- | --- | --- | --- | --- | --- | --- | --- | --- | --- | --- | --- | --- | --- | --- | --- | --- | --- | --- | --- | --- | --- | --- | --- | --- | --- | --- | --- | --- | --- | --- | --- | --- | --- | --- | --- | --- | --- | --- | --- | --- | --- | --- | --- | --- |
|  | **Non-COPD** | | **COPD** | | **Non-COPD** | | **COPD** | | **Non-COPD** | | **COPD** | | **Non-COPD** | | **COPD** | | **Non-COPD** | | **COPD** | | **Non-COPD** | | **COPD** | | **Non-COPD** | | **COPD** | | **Non-COPD** | | **COPD** | | **Non-COPD** | | **COPD** | | **Non-COPD** | | **COPD** | | **Non-COPD** | | **COPD** | |
| **Number of comorbidities** | **n** | **%** | **n** | **%** | **n** | **%** | **n** | **%** | **n** | **%** | **n** | **%** | **n** | **%** | **n** | **%** | **n** | **%** | **n** | **%** | **n** | **%** | **n** | **%** | **n** | **%** | **n** | **%** | **n** | **%** | **n** | **%** | **n** | **%** | **n** | **%** | **n** | **%** | **n** | **%** | **n** | **%** | **n** | **%** |
| **0** | 560 | 50.0 | 95 | 18.0 | 432 | 41.6 | 134 | 14.9 | 302 | 32.1 | 175 | 11.8 | 515 | 23.0 | 165 | 8.3 | 423 | 17.6 | 147 | 5.2 | 616 | 12.2 | 128 | 3.6 | 376 | 9.1 | 116 | 3.0 | 411 | 9.1 | 110 | 2.2 | 321 | 9.4 | 63 | 1.5 | 289 | 14.8 | 32 | 1.4 | 222 | 28.1 | 22 | 2.4 |
| **1** | 278 | 24.8 | 138 | 26.1 | 292 | 28.1 | 167 | 18.6 | 222 | 23.6 | 260 | 17.5 | 500 | 22.3 | 324 | 16.2 | 468 | 19.5 | 340 | 12.0 | 749 | 14.9 | 337 | 9.4 | 494 | 11.9 | 282 | 7.2 | 438 | 9.6 | 271 | 5.4 | 265 | 7.8 | 216 | 5.2 | 162 | 8.3 | 126 | 5.4 | 82 | 10.4 | 49 | 5.4 |
| **2** | 166 | 14.8 | 109 | 20.6 | 145 | 14.0 | 156 | 17.4 | 170 | 18.1 | 286 | 19.3 | 437 | 19.5 | 326 | 16.3 | 470 | 19.6 | 439 | 15.5 | 962 | 19.1 | 459 | 12.9 | 657 | 15.9 | 477 | 12.2 | 622 | 13.7 | 468 | 9.4 | 450 | 13.2 | 378 | 9.0 | 237 | 12.1 | 199 | 8.5 | 110 | 13.9 | 108 | 11.9 |
| **3** | 66 | 5.9 | 72 | 13.6 | 86 | 8.3 | 148 | 16.5 | 106 | 11.3 | 235 | 15.8 | 336 | 15.0 | 334 | 16.7 | 380 | 15.9 | 466 | 16.5 | 900 | 17.9 | 547 | 15.3 | 719 | 17.3 | 534 | 13.6 | 728 | 16.0 | 658 | 13.2 | 546 | 16.1 | 459 | 11.0 | 272 | 13.9 | 307 | 13.1 | 98 | 12.4 | 126 | 13.9 |
| **4** | 36 | 3.2 | 38 | 7.2 | 43 | 4.1 | 107 | 11.9 | 70 | 7.4 | 187 | 12.6 | 199 | 8.9 | 264 | 13.2 | 275 | 11.5 | 395 | 14.0 | 680 | 13.5 | 504 | 14.1 | 633 | 15.3 | 563 | 14.4 | 684 | 15.1 | 716 | 14.4 | 531 | 15.6 | 588 | 14.1 | 304 | 15.5 | 313 | 13.4 | 97 | 12.3 | 135 | 14.9 |
| **5** | 7 | 0.6 | 29 | 5.5 | 23 | 2.2 | 63 | 7.0 | 39 | 4.1 | 113 | 7.6 | 119 | 5.3 | 201 | 10.1 | 173 | 7.2 | 311 | 11.0 | 456 | 9.1 | 434 | 12.2 | 455 | 11.0 | 476 | 12.2 | 572 | 12.6 | 607 | 12.2 | 443 | 13.0 | 534 | 12.8 | 236 | 12.1 | 294 | 12.6 | 70 | 8.9 | 97 | 10.7 |
| **6** | 3 | 0.3 | 13 | 2.5 | 7 | 0.7 | 40 | 4.5 | 16 | 1.7 | 73 | 4.9 | 58 | 2.6 | 125 | 6.3 | 91 | 3.8 | 217 | 7.7 | 283 | 5.6 | 349 | 9.8 | 304 | 7.3 | 387 | 9.9 | 388 | 8.5 | 493 | 9.9 | 249 | 7.3 | 435 | 10.4 | 146 | 7.5 | 214 | 9.2 | 45 | 5.7 | 93 | 10.3 |
| **7** | 1 | 0.1 | 11 | 2.1 | 5 | 0.5 | 25 | 2.8 | 6 | 0.6 | 54 | 3.6 | 39 | 1.7 | 76 | 3.8 | 58 | 2.4 | 138 | 4.9 | 169 | 3.4 | 243 | 6.8 | 180 | 4.3 | 271 | 6.9 | 270 | 5.9 | 427 | 8.6 | 215 | 6.3 | 385 | 9.2 | 119 | 6.1 | 197 | 8.4 | 30 | 3.8 | 84 | 9.3 |
| **8** | 2 | 0.2 | 23 | 4.4 | 6 | 0.6 | 57 | 6.4 | 10 | 1.1 | 102 | 6.9 | 38 | 1.7 | 182 | 9.1 | 59 | 2.5 | 377 | 13.3 | 217 | 4.3 | 567 | 15.9 | 327 | 7.9 | 811 | 20.7 | 427 | 9.4 | 1224 | 24.6 | 378 | 11.1 | 1122 | 26.8 | 190 | 9.7 | 653 | 28.0 | 36 | 4.6 | 192 | 21.2 |
| **% of individuals with 2**  **or more comorbidities** |  | **25.1** |  | **55.9** |  | **30.3** |  | **66.4** |  | **44.3** |  | **70.7** |  | **54.7** |  | **75.5** |  | **62.8** |  | **82.8** |  | **72.9** |  | **87.0** |  | **79.0** |  | **89.8** |  | **81.3** |  | **92.3** |  | **82.8** |  | **93.3** |  | **76.9** |  | **93.2** |  | **61.5** |  |  |

**S2 Table. Number and percentage of individuals in each age categories distributed to their comorbidities load (0 to ≥8 comorbidities)**
