# Supplementary material for: Chronic Obstructive Pulmonary Disease (COPD) as a disease of early aging: Evidence from the EpiChron Cohort
Source: PLoS One. 2018 Feb 22;13(2):e0193143. doi: 10.1371/journal.pone.0193143 (PMC5823454; doi:10.1371/journal.pone.0193143)
Supplement: S3 Table — (DOCX) [file pone.0193143.s013.docx]

**S2 Table. Smokers subset characteristics**

| **Demographics** | **COPD** | **Controls** | **p-value** |
| --- | --- | --- | --- |
| n | 1,914 | 1,914 |  |
| Age years (mean, SD) | 63 (±11) | 63 (±11) |  |
| **Age brackets distribution (n, % of total for the group)** | | | |
| Age ≤ 55 years | 482 (25%) | 443 (23%) |  |
| Age >55 and <65 | 468 (25%) | 507 (27%) |  |
| Age ≥ 65 years | 964 (50%) | 964 (50%) |  |
| **Gender and smoking status (n, % of total for the group)** | | | |
| Male n (%) | 1,608 (84%) | 1,608 (84%) |  |
| Female n (%) | 306 (16%) | 306 (16%) |  |
| **Number of Comorbidities/ patient (Mean, SD, 95% CI)** | | | |
| Comorbidities (whole) | 4.5 ± 3.3, (4.3-4.6) | 3.3± 2.7, (3.1-3.4) | <0.001 |
| Male | 4.5± 3.3, (4.4-4.7) | 3.3± 2.7, (3.2-3.4) | <0.001 |
| Female | 4.2± 3.2, (3.9-4.6) | 3.1± 2.8, (2.8-3.4) | <0.001 |
| **3-year Mortality** | | | |
| Number of death (%) | 269 (14.0 %) | 141 (7.37 %) | <0.001 |
